# Anticipatory Smooth Eye Movements in Autism Spectrum Disorder

**DOI:** 10.1371/journal.pone.0083230

**Published:** 2013-12-23

**Authors:** Cordelia D. Aitkin, Elio M. Santos, Eileen Kowler

**Affiliations:** Department of Psychology, Rutgers University, Piscataway, New Jersey, United States of America; Barrow Neurological Institute, United States of America

## Abstract

Smooth pursuit eye movements are important for vision because they maintain the line of sight on targets that move smoothly within the visual field. Smooth pursuit is driven by neural representations of motion, including a surprisingly strong influence of high-level signals representing expected motion. We studied anticipatory smooth eye movements (defined as smooth eye movements in the direction of expected future motion) produced by salient visual cues in a group of high-functioning observers with Autism Spectrum Disorder (ASD), a condition that has been associated with difficulties in either generating predictions, or translating predictions into effective motor commands. Eye movements were recorded while participants pursued the motion of a disc that moved within an outline drawing of an inverted Y-shaped tube. The cue to the motion path was a visual barrier that blocked the untraveled branch (right or left) of the tube. ASD participants showed strong anticipatory smooth eye movements whose velocity was the same as that of a group of neurotypical participants. Anticipatory smooth eye movements appeared on the very first cued trial, indicating that trial-by-trial learning was not responsible for the responses. These results are significant because they show that anticipatory capacities are intact in high-functioning ASD in cases where the cue to the motion path is highly salient and unambiguous. Once the ability to generate anticipatory pursuit is demonstrated, the study of the anticipatory responses with a variety of types of cues provides a window into the perceptual or cognitive processes that underlie the interpretation of events in natural environments or social situations.

## Introduction

Smooth pursuit eye movements perform an important function in vision by allowing the line of sight to track selected objects that move smoothly through the environment. Unlike saccadic eye movements, which can be used to look in any chosen direction, regardless of the contents of the visual scene, it is not possible to initiate smooth pursuit across a stationary visual scene at will. The initiation and control of smooth pursuit requires signals representing motion. The types of motion signals that contribute to the initiation and control of smooth pursuit are varied and complex, and include the motion of isolated objects, global motion of fields of random dots, second order motion, illusions of motion, and, of most relevance to the present study, expectations of motion. For reviews, see [1]–[5].

Indications that expected motion plays a role in the control of smooth pursuit go back to some of the earliest studies of pursuit, in which the targets to be tracked moved in periodic, repetitive patterns [6]–[8]. Pursuit showed surprisingly short sensorimotor delays, with the motion of the eye often matching the motion of the target nearly perfectly, sometimes even changing direction in advance of the target. By contrast, pursuit of targets moving randomly is quite poor [9]. The ability to overcome sensorimotor delays during pursuit of periodic motions was initially attributed to learning, specifically, to the ability of the pursuit control mechanisms to generate patterns of smooth eye movements to match target motions tracked in the recent past [6]–[8], [10]. More recent evidence from a variety of experimental paradigms that supports the ability of pursuit to reproduce patterns of target motion after minimal exposure, pointing to a role for learning and short-term memory in generating pursuit responses [11]–[13].

Anticipatory pursuit, however, is not just a matter of learning to reproduce motion patterns seen or tracked in the recent past [14]. Visual or auditory cues that signal the direction of future target motion are able to generate vigorous anticipatory smooth pursuit in the direction of the expected future motion of a target, either prior to motion onset [14]–[19], or prior to an expected change in motion direction [20]–[24]. These results point to an involvement of neural processes that are able to transform representations of expected motion into smooth oculomotor commands. Two plausible cortical neural areas that could be involved are the medial temporal area (MT) and dorsomedial frontal cortex (DMFC). Both have been found to be sensitive to cues that signal future motion or future events [25]–[28]. The involvement of DMFC is supported further by findings that supplementary eye field (a part of DMFC) shows activity patterns that correlate strongly with anticipatory smooth pursuit [29]–[32].

Given the importance of anticipatory behavior for overcoming sensorimotor delays, the study of anticipatory pursuit could provide an effective way to understand neural processes responsible for interpreting cues, generating predictions, and transforming predictions into motor commands in both typical and disordered neural systems. One important example is Autism Spectrum Disorder (ASD), a complex neurogenetic disorder characterized by social, perceptual, sensorimotor and cognitive deficits; see, for example [33].

ASD has been associated with impairments in the ability to generate and use predictions in motor tasks, including smooth pursuit. Takarae, Minshew, Luna, Krisky, & Sweeney [34], for example, found that the gain of pursuit (ratio of eye velocity/target velocity) of both constant velocity and oscillating target motions was about 10% lower in individuals with ASD than in typical individuals [35]. Takarae et al. [34] attributed the reduced gain to sensorimotor deficiencies, as well as to possible deficiencies in generating predictive responses. A subsequent study of neural activity during pursuit of predictable motions supported these conclusions by showing that those with ASD had lower levels of activity in cortical areas, such as frontal eye fields, parietal cortex and dorsolateral prefrontal cortex, which have been associated with the contribution of high level factors (anticipation, learning and attention) to eye movements [36].

Other studies found results that were consistent with the possibility that difficulties in forming predictions, or in generating anticipatory motor behavior, may be associated with ASD. For example, ASD subjects made fewer anticipatory saccades than typical subjects when following the motion of a target that jumped back and forth [37]. Gomot & Wicker [38] proposed that ASD may be characterized by a difficulty in generating predictions from contextual cues, while Gowan & Hamilton [39] raised the possibility that generating feed-forward predictions of the sensory consequences of ones' own movement, widely seen as an important aspect of fluid and effective motor behavior, might be impaired in ASD.

Gowan & Hamilton [39] also noted that definitive empirical distinctions between the role of anticipation and the role of sensorimotor processes are often difficult to achieve, raising questions about whether, or to what degree, anticipatory abilities account for any motor impairments observed in ASD. Falck-Ytter [40], for example, using a task with minimal motor demands, found that children with ASD were not different from neurotypicals in generating the appropriate anticipatory saccadic eye movements when watching a movie of objects being manipulated by actors. Such findings, when viewed along with prior findings suggesting impairments in predictive motor behaviors in ASD, indicate that the ability of those with ASD to generate anticipatory motor behaviors is not resolved. A study of anticipatory smooth pursuit eye movements could be valuable in providing an unambiguous estimation of predictive abilities in ASD.

The present study examined anticipatory smooth pursuit eye movements in a group of participants with high-functioning ASD. The anticipatory smooth eye movements were elicited by visual cues that were incorporated directly into the display. These cues consisted of a visual symbol that provided definitive and perceptually salient information about the future path of motion of the target. Perceptually salient, symbolic visual cues that reveal the motion pathway are known to be highly effective in generating anticipatory smooth eye movements with no special task instructions in neurotypical individuals [19], [21], [23], [24].

Any study of smooth pursuit in ASD has to take into account the possibility that performance could be affected by deficits in either motion processing or low-level aspects of oculomotor control. Processing of motion in ASD has been investigated extensively using both psychophysical and neurophysiological measures; for reviews, see [41], [42]. These reviews, along with more recent results, argue that a principal difficulty in ASD is the integration of motion signals over space and time. Brieber et al. [43], for example, found that differences in neural activity in areas such as V5 and parietal cortex between random and coherent motion stimuli were smaller in ASD participants than in control participants. Robertson et al. [44] found that ASD subjects performed more poorly than typicals when identifying the predominant direction of motion of fields of random dots with varying coherence levels, but only when stimulus duration was brief (< about 0.5 s). Foss-Fieg [45] reported enhancements of discrimination of motion direction with high-contrast stimuli in ASD relative to typicals, a pattern of results suggestive of a possible abnormal balance between excitatory and inhibitory processes in ASD. In addition, the possibility of some degree of general oculomotor dysfunction in ASD has been suggested both by observations of lower pursuit gain (ratio of eye velocity/target velocity) during all but the initial phases of pursuit of both constant velocity and oscillating motions [34], as well as by findings that those with ASD often make larger and more frequent saccades in a variety of visual tasks [33], [35], [46], [47].

Such deficits in low-level processing, either motion processing or oculomotor control, are not likely to be responsible for any specific impairment in anticipatory smooth eye movements in ASD. This is because the effects of anticipation and of lower-level sensorimotor processes are opposite to one another during pursuit. Lower-level oculomotor processes act to keep the line of sight near the target, minimizing the speed of the target on the retina. Anticipatory smooth eye movements, on the other hand, found shortly before a change in the direction of target motion, briefly take the line of sight away from the moving target, producing momentary increases in the speed of the target on the retina. Thus, finding a deficiency in generating anticipatory smooth eye movements would suggest a specific deficit of the neural circuitry responsible either for generating the predictions about the path of future target motion, or for conveying the predictions downstream within the pursuit system.

## Experimental Methods

### Ethics

Written informed consent was obtained from each participant prior to participation. The research protocol was approved by the Rutgers University Institutional Review Board for the Protection of Human Subjects and is in accordance with the Declaration of Helsinki.

### Participants

Ten volunteers with high-functioning Autism Spectrum Disorder (ASD) (2 female) and ten neurotypical participants (2 female) were tested. (An additional 4 individuals, 1 ASD and 3 typical, visited the lab for testing, however, a suitable signal could not be obtained from the eye tracker due to eyelids obscuring a portion of the pupil.)

All participants were over 18 years old (average age of ASD participants  = 25 years, SD  = 3 years 4 months; average age of typical participants  = 19 years 10 months, SD = 1 year 4 months), and were not told the purpose of the experiment, beyond that it was a study of eye movements. The ASD participants were recruited through mailings to local support groups. Diagnoses of Autism or Autism Spectrum Disorder were verified by administration of the Autism Diagnostic Observation Schedule. Cognitive abilities of the ASD group were adequate to provide informed consent and to understand the simple task instructions (scores on the Wechsler Abbreviated Scale of Intelligence ranged from 81 to 119 for 8 of the ten ASD participants; WASI's were not given to the remaining two participants, who had completed college). Participants in the typical group were drawn from the Rutgers University Department of Psychology participant pool. All testing was done within a single visit to the laboratory, which lasted approximately 1 hour. ASD participants were monetarily compensated for their time. The Psychology students received course credit.

### Eye movement recording

Eye movements (right eye only) were recorded by the Eyelink 1000 (SR Research, Osgoode, Canada), tower-mounted version, sampling at 1000 Hz. Chin and forehead supports were used to stabilize the head. Viewing was binocular.

### Stimulus and materials

The stimulus was displayed in a fully-lighted room on a Viewsonic G90fB 19″ CRT monitor, 1024×768 resolution, refresh rate 60 Hz, viewed from a distance of 118 cm. The display area subtended 16.2° (972 min arc) horizontally by 12.3° (738 min arc) vertically.

**Figure 1:**
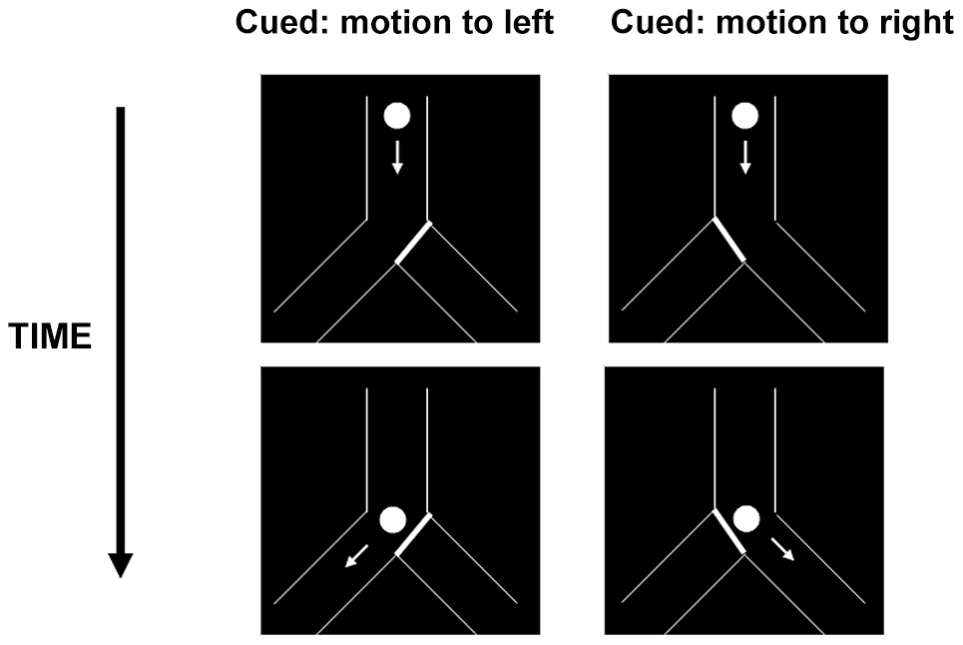
Stimulus Display for Cued Target Motion. The disc moves down the tube and then travels down whichever branch (left or right) is not blocked by the visual barrier cue.

The display used to study anticipatory smooth eye movements contained a line drawing of an inverted Y-shaped tube (tube arm width 68 min arc) (Figure 1). The walls of the tube and the disc were white, displayed on a black background. The oblique branches of the “Y” were at a directional angle of 40° from vertical. A 58 min arc diam disc, initially located near the top of the tube, moved downward for a vertical distance of 532 min arc at a speed of 174 min arc/s, and then traveled down either right or left oblique branch. The horizontal component of the velocity in the oblique branch was 114 min arc/s, and the total vector velocity of the disc remained the same throughout the trial. This velocity is well over threshold to activate pursuit (e.g., [48]) while minimizing potential distractions due to noticeable position or velocity errors that are inevitable when more rapid target motions are tracked with even very high pursuit gains.

Two conditions were tested. In the *cued condition* the path of the target was indicated by a solid white line “barrier” cue (see Fig. 1) that blocked the untraveled branch. In the *uncued condition* no barrier cue was shown. In both conditions, the path taken by the disc, right or left, was randomly selected prior to the trial, with each side having an equal probability of being chosen.

### Initial familiarization and initial calibration

Procedures were formulated with the goal of maximizing the comfort level of the ASD participants. Four of the 10 ASD participants were accompanied by a family member who remained in the testing room during the study. Before testing, participants were given ample time to tour the lab, discuss the operation of the eyetracker, and read and sign consent forms.

### Experimental sessions

Testing was carried out in a series of brief experimental sessions containing no more than ten trials each. The nine-point calibration incorporated into Eyelink's software was run prior to each of the experimental sessions. Between sessions subjects took brief breaks of about 3 to 5 minutes.

The experimental trials were each started by the participant when ready by pressing a button on the game pad. Additionally, the participants were asked to blink a few times before starting each trial, to minimize the number of blinks during the trial.

### Fixation, saccades and pursuit

The first experimental session (5 trials) examined eye movements during maintained fixation of a central, stationary target (35 min arc x 35 min arc cross). The participant was asked to look at the cross, start the trial when ready by pressing a button on a game pad, and to continue looking at the cross throughout the 5s trial. After each trial, the cross briefly disappeared to signal that a new trial could be initiated.

The next two sessions tested two different simple eye movement tasks solely for the purpose of further familiarizing participants with the routine of experimental testing with targets that would be in motion. The first of these two sessions (5 trials) consisted of a simple saccadic task in which the central fixation cross jumped 168 min arc either to the right or to the left. Participants were asked to shift gaze to the cross after it jumped. The second session (3 trials) consisted of the cross changing to a disc at trial onset and then moving smoothly 174 min arc/s to the right or to the left. Participants were asked to pay attention to the motion, as in Kowler [23] and Santos et al. [19]. The experimenters examined the on-line display of eye position to verify that smooth pursuit eye movements occurred and that the participants were not looking away from the disc to other regions of the display or room. All participants were able to use saccades to follow the jumps of the cross, and smooth pursuit to follow the smooth motions of the disc. The data from this preliminary testing were not analyzed because the purpose was solely to establish familiarization and willingness to cooperate with the experimental procedures.

### Procedures for testing anticipatory smooth eye movements

The remaining experimental sessions measured smooth pursuit with the stimulus configuration shown in Fig. 1 (the barrier cue shown in the figure was present only in the cued condition). Most of the participants ran in 8 sessions, 4 for the cued condition and 4 for the uncued condition, 10 trials each. The exceptions were participant ASD5, who ran in 4 sessions (2 for each cuing condition), and ASD9, who ran 12 sessions (6 for each cuing condition). Sessions with or without the barrier cue were alternated, beginning with a session without the cue.

Before each trial the entire stimulus, consisting of the outline drawing of the tube, the cross at the top of the tube, and (for the cued condition) the barrier, was shown. The participant fixated the cross and pressed a button on the game pad when ready to start the trial. At this point, the cross changed to the disc. One second later the disc began to move down the tube. The instructions were to pay attention to the motion of the disc. Occasionally, a participant in either group would use a saccadic eye movement to jump ahead of the moving disc and look toward the choice point in the Y-shaped tube. If this occurred, participants were reminded to pay attention to the motion and asked not to jump ahead of the disc.

**Figure 2:**
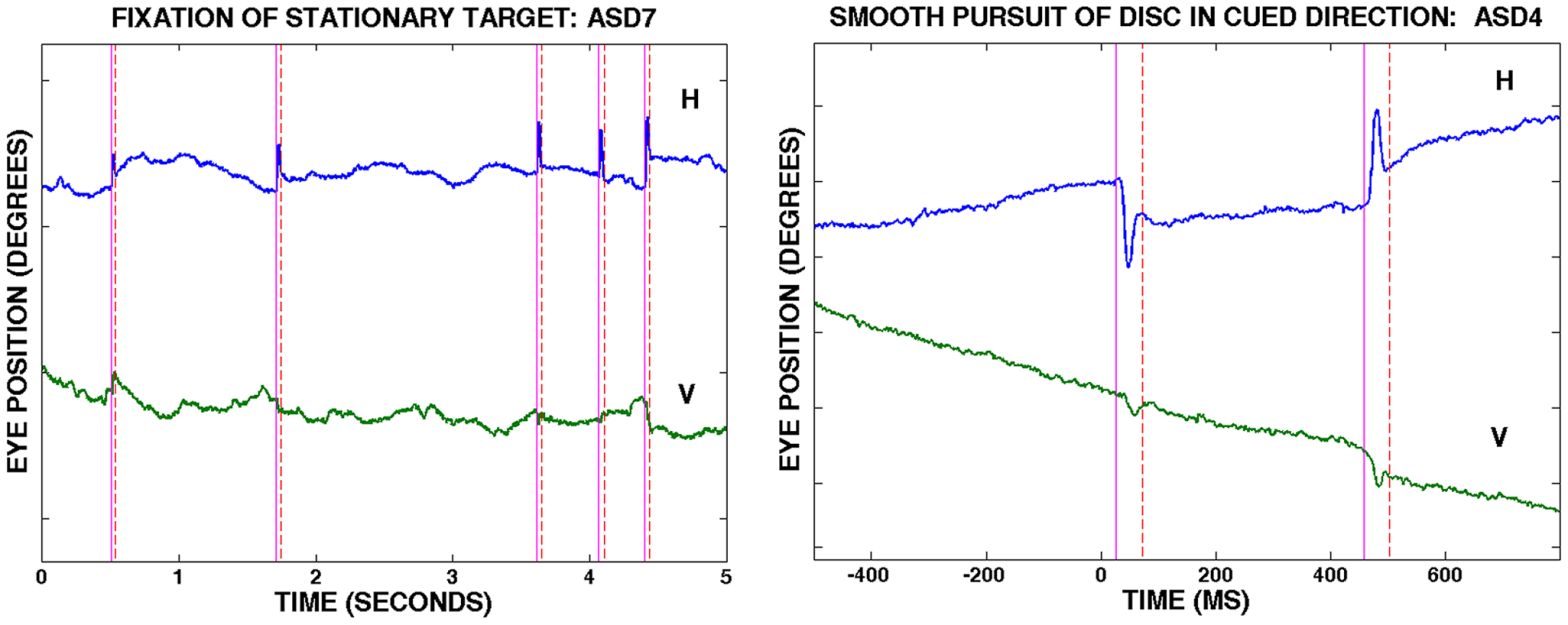
Representative Eye Traces During Fixation and During Smooth Pursuit. (Left) Representative record showing horizontal (H) and vertical (V) eye movements over time during 5 seconds of fixation of the stationary target. Participant ASD7. (Right) Representative record showing horizontal (H) and vertical (V) eye movements during 1.3 seconds of smooth pursuit of the disc moving down the tube; cued condition. The disc entered the right oblique branch at time  =  0. Participant ASD4. The vertical lines represent computer-generated markers showing beginning and end of saccades. Upward deflections indicate movements to the right or up. The separation between tic marks on the ordinate are 1 degree (60 min arc). Horizontal anticipatory smooth eye movements to the right, in the direction of cued motion, can be seen prior to time = 0 in the graph on the right.

*Analysis.* The onsets and offsets of saccades were determined offline by computing eye velocity during 13 ms samples, with onsets separated by 1 ms. Saccade onset and offsets were detected using a velocity criterion. The criterion was determined and subsequently confirmed for each subject based on an exhaustive examination of analog records of eye position (see example in Fig. 2). Criteria (eye velocity during 13 ms intervals) ranged from 7–16 deg/s (420–960 min arc/s). Determination of saccade offsets were subjected to the additional constraint that velocity had to be below the criterion for 33 ms, which was long enough to bypass the overshoots typically accompanying saccades. For the determination of smooth pursuit velocity, eye velocity was computed for successive 50 ms intervals whose onsets were separated by 2 ms. Intervals containing saccades or blinks were discarded. Eye velocity was also calculated for longer intervals, namely: (1) from 150 ms before to 50 ms after the onset of horizontal motion (where the onset of horizontal motion is the time when the disc entered an oblique branch), and (2) from 400 ms to 800 ms after the onset of horizontal target motion. The former interval was used to assess the velocity of the anticipatory response while the latter was used to obtain a measure of maintained pursuit, where maintained pursuit refers to the interval after eye velocity had sufficient time to reach values near the velocity of the target. Velocity intervals containing saccades, blinks or episodes in which the signal from the tracker was lost due to interference from the eyelid were discarded.

## Results

### Anticipatory smooth eye movements

**Figure 3:**
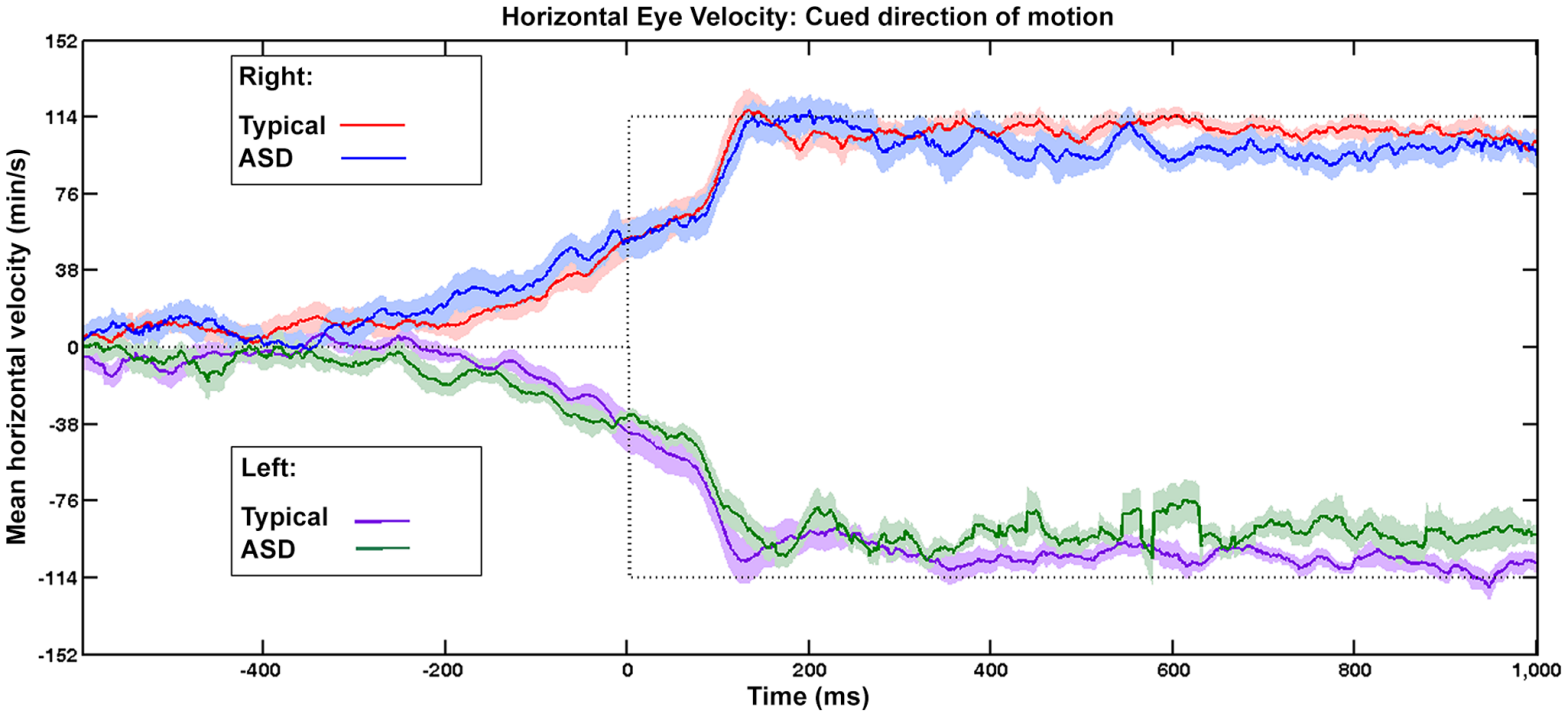
Mean Eye Velocity Over Time, Motion in Cued Directions. Mean horizontal eye velocity (average of participant means) when the visual barrier cue indicated the future path of the moving disc (left or right), for ASD (blue and green lines) and typical (red and purple lines) participants. The dotted line indicates the horizontal velocity of the disc. The disc entered the oblique branch at time  = 0 and traveled either to the right (positive values) or left (negative values) Shading indicates +/− Standard Error.

**Figure 4:**
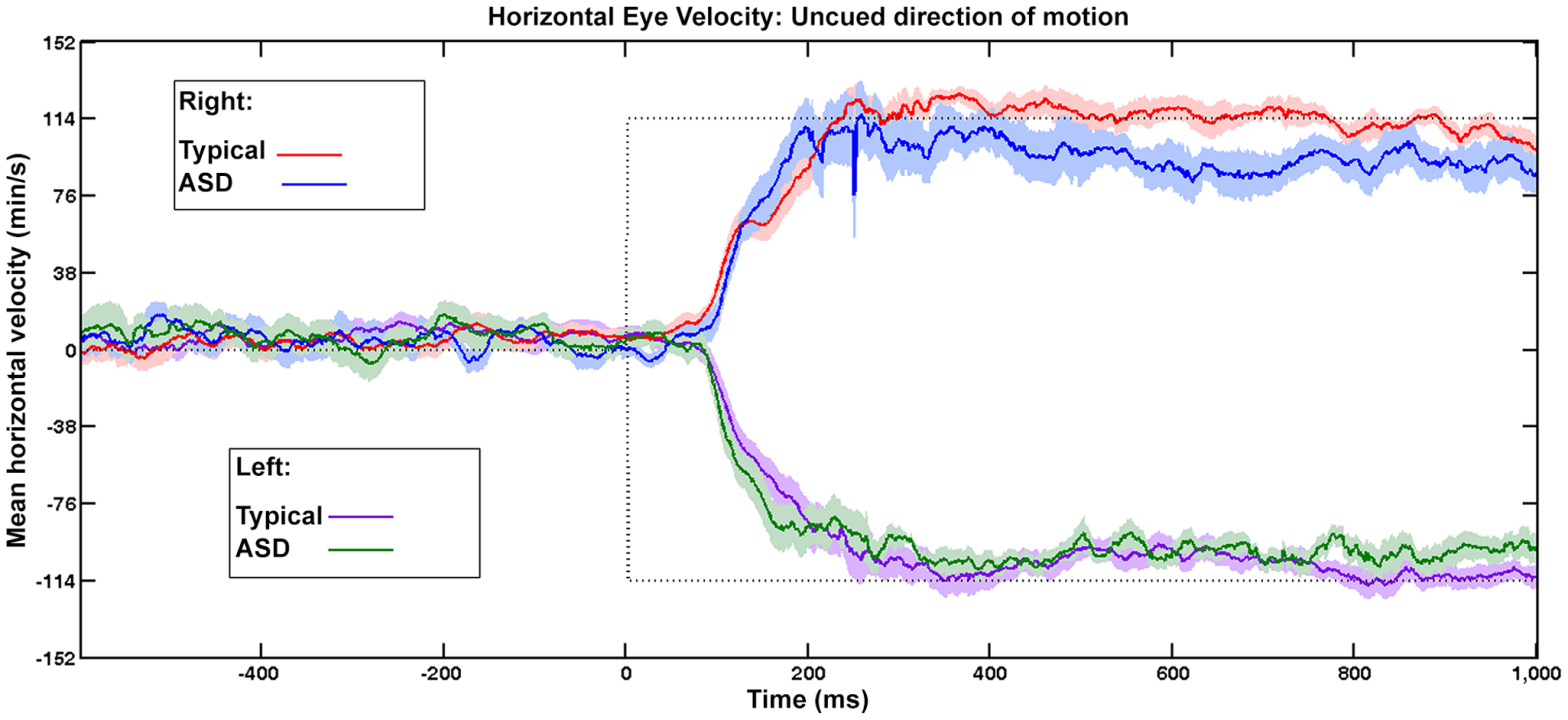
Mean Eye Velocity Over Time, Motion in Uncued Directions. Mean horizontal eye velocity (average of participant means) when no cue indicated the future path of the moving disc (left or right), for ASD (blue and green lines) and typical (red and purple lines) participants. The dotted line indicates the horizontal velocity of the disc. The disc entered the oblique branch at time  = 0 and traveled either to the right (positive values) or left (negative values) Shading indicates +/− Standard Error.

Anticipatory smooth eye movements were found in those with ASD. A sample eye trace showing anticipatory smooth eye movements in an individual with ASD in the cued condition is shown in Fig. 2. Figure 3 shows mean horizontal eye velocity (average of participant means) over time for the cued condition. Note that zero on the abscissa represents the onset of horizontal target motion, when the disc entered an oblique branch. Anticipatory smooth eye movements (eye velocity in the direction of the expected motion of the target) can be seen in both ASD and typical groups before the onset of horizontal target motion, and reached a velocity of about half that of the target by the time horizontal target motion began. When the cue was not present (Fig 4), pursuit did not begin until at least 100 ms after the onset of horizontal target motion.

Statistical analyses confirmed the existence of anticipatory smooth eye movements. An ANOVA was performed on the mean horizontal eye velocities (50 ms samples; see methods) at the time of onset of horizontal target motion (time = 0 in Fig. 3). The ANOVA used direction (left vs. right), participant group (ASD vs. typical), and cuing condition (uncued vs. cued) as grouping variables. There was a significant main effect of direction (*F* (1,72) = 84.1, *p*<<0.001), due principally to the results in the cued condition, and a significant interaction between direction and cuing (*F*(1,72) = 97.3, *p*<<0.001). Post-hoc tests confirmed that the interaction was due to a significant difference in velocity between left and right directions in the cued condition; and a significant difference between the velocities in cued-left vs. uncued-left conditions, and between the cued-right and uncued-right conditions (see Table 1).

**Table 1:** ANOVA statistics on horizontal eye velocities at the onset of horizontal target motion.

Source	Sum of Squares	df	Mean Squares	*F*	*p*
**Direction of Target**	66798.1	1	66798.1	84.10	0.0000
**Cued/Not Cued**	347.8	1	347.8	0.44	0.5103
**ASD/Neurotypical**	0.6	1	0.6	0.00	0.9773
**Direction by Cueing**	77258.7	1	77258.7	97.27	0.0000
**Direction by Condition**	357.4	1	357.4	0.45	0.5045
**Cueing by Condition**	413.6	1	413.6	0.52	0.4729
**Direction by Cueing by Condition**	104.0	1	104.0	0.13	0.7186
**Error**	57189.7	72	794.3		
**Total**	202469.8	79			

In addition to the finding of anticipatory smooth eye movements in both groups, the statistical analyses also verified that there were no significant differences between anticipatory eye velocities in the ASD and typical groups. This supports the central finding of this study, namely, that anticipatory smooth eye movements in high-functioning ASD are not different from those in typical populations.

Anticipatory smooth eye movements were present in each participant. Table 2 shows average eye velocity for each participant in the cued condition, computed over the interval beginning 150 before the onset of horizontal target motion and ending 50 ms after the onset of horizontal target motion. Each participant shows eye velocities in the direction of the cue. While the average velocity of the ASD participants was faster than that of the typical participants (see also Fig. 3) differences between groups on the results in Table 2 did not reach significance. (p = .27, df = 775, t = 1.4, two-tailed.).

**Table 2:** Mean horizontal velocity (min arc/s) of anticipatory pursuit for individual participants: Cued Condition.

*ASD*					*Neurotypical*				
	Cued Motion to Left		Cued Motion to Right			Cued Motion to Left		Cued Motion to Right	
Participant	Mean (SD)	N	Mean (SD)	N	Participant	Mean (SD)	N	Mean (SD)	N
ASD1	−33 (40)	7	19 (59)	18	TYP1	−2 (32)	22	6 (39)	18
ASD2	−15 (32)	15	47 (27)	25	TYP2	−25 (45)	22	26 (28)	18
ASD3	−25 (21)	20	36 (32)	20	TYP3	−17 (28)	21	50 (34)	19
ASD4	−21 (34)	21	42 (43)	14	TYP4	−37 (26)	19	58 (29)	21
ASD5	−27 (39)	12	93 (29)	8	TYP5	−6 (31)	23	25 (29)	17
ASD6	−6 (47)	20	28 (39)	18	TYP6	10 (31)	16	28 (32)	24
ASD7	−27 (36)	23	40 (23)	17	TYP7	−37 (31)	22	10 (28)	18
ASD8	−26 (42)	16	45 (33)	24	TYP8	−27 (31)	20	24 (40)	20
ASD9	−41 (35)	26	20 (35)	34	TYP9	−26 (22)	27	57 (26)	13
ASD10	−31 (56)	15	24 (37)	23	TYP10	−34 (28)	28	21 (23)	12
Group Mean	−25 (10)		39 (22)			−20 (16)		31 (18)	

Eye velocities were taken over the interval from 150 ms before to 50 ms after the onset of horizontal target motion. Positive values represent eye velocities to the right; negative values to the left. The maximum number of trials/participant  = N(left)+N(right) = 40, except for ASD5 who was tested in 20 trials, and ASD9 who was tested in 60 trials

### The very first trial tracking motion in cued directions

A fundamental question about anticipatory pursuit responses is whether the anticipatory movements elicited by the cues resulted from learning that had occurred within the laboratory, or, alternatively, from learning that occurred due to exposure to cues in the natural environment. Symbolic cues, such as the barriers used in the present study, could have acquired the power to generate anticipatory smooth pursuit before the participant ever saw our laboratory stimuli because these cues are semantically consistent with the pathway of motion of the target. To address the question of whether the relevant learning about the cues occurred during or prior to the visit to the lab, eye velocity during the anticipatory interval (150 ms before to 50 ms after the onset of horizontal motion) was examined for the very first trial each participant ran in the cued condition.

Table 3 shows eye velocity during the anticipatory interval for each participant during his or her very first cued trial, organized according to the direction of motion signaled by the cue (right or left) in the first trial. Fifteen of the 20 participants (8 of the 10 ASD participants, and 7 of the 10 typical participants) showed eye velocity in the cued direction on the first trial. The difference between anticipatory eye velocity when the cue in the first trial indicated motion to the right vs. motion to the left was significant (t = 2.58, df = 16, p = .02, two-tailed). These results show that any learning needed to generate anticipatory pursuit responses to the cues, in either ASD or typical participants, occurred prior to the lab visit, due to experience in the natural environment. Learning or practice within the experimental sessions was not needed to generate the anticipatory pursuit responses.

**Table 3:** Horizontal eye velocity (min arc/s) during first trials in the cued condition.

*ASD*				*Neurotypical*			
Cued Motion to Left		Cued Motion to Right		Cued Motion to Left		Cued Motion to Right	
Participant	Eye Velocity (minarc/s)	Participant	Eye Velocity (minarc/s)	Participant	Eye Velocity (minarc/s)	Participant	Eye Velocity (minarc/s)
ASD4	−11	ASD1	20	TYP2	47	TYP1	−8
ASD5	−42	ASD2	62	TYP3	−1	TYP4	81
ASD6	66	ASD3	98	TYP6	17	TYP5	24
ASD7	−57	ASD10	15	TYP8	−40	TYP7	10
ASD8	−75			TYP9	−56		
ASD9	9			TYP10	−59		
Group Mean (SD)	−18 (51)		49 (39)		−15 (43)		27 (38)

Eye velocities were taken over the interval from 150 ms before to 50 ms after the onset of horizontal target motion. Positive values represent eye velocities to the right, negative to the left.

### Other aspects of smooth pursuit

#### Eye velocities during maintained pursuit

Examination of Figures 3 and 4 shows that once pursuit was well underway, and the eye velocity reached values close to the velocity of the targets (about 300 ms after the onset of horizontal motion), the average eye velocity for the ASD participants was slightly slower than the average velocity for the typical participants. To examine whether there were reliable group differences in pursuit after it reached values close to the velocity of the target, average eye velocity for each participant was determined for the interval 400–800 ms after the onset of horizontal target motion. Results are shown in Tables 4 and 5 for the cued and uncued conditions, respectively. Although average pursuit velocities were generally lower for those with ASD, the group differences were only marginally significant (t = 2.0; df = 18, two-tailed, p = .046) and due mainly to the performance of one of the ASD participants (ASD1).

**Table 4:** Mean eye velocity (min arc/s) during maintained pursuit, cued condition.

*ASD*			*Neurotypical*		
Participant	Left	Right	Participant	Left	Right
	Mean (SD)	Mean (SD)		Mean (SD)	Mean (SD)
ASD1	−96 (56)	68 (68)	TYP1	−77 (32)	77 (30)
ASD2	−79 (25)	110 (23)	TYP2	−83 (32)	116 (37)
ASD3	−92 (29)	84 (25)	TYP3	−112 (23)	114 (30)
ASD4	−69 (49)	94 (38)	TYP4	−103 (26)	94 (27)
ASD5	−92 (31)	119 (25)	TYP5	−93 (19)	97 (22)
ASD6	−88 (47)	82 (39)	TYP6	−89 (34)	117 (28)
ASD7	−88 (31)	95 (15)	TYP7	−92 (19)	91 (25)
ASD8	−103 (23)	109 (22)	TYP8	−107 (36)	99 (38)
ASD9	−82 (37)	75 (40)	TYP9	−92 (18)	104 (30)
ASD10	−90 (21)	85 (33)	TYP10	−93 (34)	83 (30)
Group Mean (SD)	−88 (9)	92 (16)		−94 (11)	99 (14)

Eye velocities were taken over the interval from 400 ms to 800 ms after the onset of horizontal target motion to the left or to the right. Positive values represent eye velocities to the right, negative to the left.

**Table 5:** Mean eye velocity (min arc/s) during maintained pursuit, uncued condition.

*ASD*			*Neurotypical*		
Participant	Left	Right	Participant	Left	Right
	Mean (SD)	Mean (SD)		Mean (SD)	Mean (SD)
ASD1	−116 (58)	14 (66)	TYP1	−77 (33)	71 (33)
ASD2	−84 (22)	114 (27)	TYP2	−84 (28)	112 (33)
ASD3	−114 (21)	88 (33)	TYP3	−99 (25)	111 (23)
ASD4	−86 (38)	96 (37)	TYP4	−113 (26)	121 (32)
ASD5	−89 (27)	115 (36)	TYP5	−91 (22)	111 (29)
ASD6	−79 (33)	87 (35)	TYP6	−87 (36)	125 (37)
ASD7	−98 (19)	108 (24)	TYP7	−101 (29)	120 (18)
ASD8	−98 (26)	95 (38)	TYP8	−93 (33)	111 (33)
ASD9	−109 (34)	70 (42)	TYP9	−107 (23)	113 (22)
ASD10	−99 (31)	92 (43)	TYP10	−78 (27)	90 (20)
Group Mean (SD)	−97 (13)	88 (29)		−93 (12)	109 (16)

Eye velocities were taken over the interval from 400 ms to 800 ms after the onset of horizontal target motion to the left or to the right. Positive values represent eye velocities to the right, negative to the left.

#### Pursuit onset latency

The latency of the smooth pursuit response to the onset of horizontal target motion was estimated only for the uncued condition. In the uncued condition the average baseline eye velocity was close to zero (Fig. 4), thus a determination of the onset would be more precise in the uncued condition than in the cued condition. The onset of pursuit for each participant was set as the first velocity sample that was more than 4 SD's greater than average baseline eye velocity, where the baseline velocity was determined by averaging all velocity samples during the 400 ms interval prior to the onset of horizontal target motion. The average latency of pursuit, defined as the difference between the time of onset of pursuit and the time of onset of horizontal target motion, was 105 ms (SD 11.4), averaged over all subjects in the typical group, and 154 ms (SD 78) in the ASD group, for rightward motion. For leftward motion the values were 122 ms (SD 35) for the typical group and 147 ms (SD 81) for the ASD group. Although average latencies for the ASD group were longer, the differences were not reliable (t = 0.9, df = 18, p = .37, two-tailed; for leftward motion; t = 2.0, df = 18, p = .065, two-tailed, for rightward motion).

#### Saccadic eye movements during smooth pursuit

Saccades, in or opposite to the direction of motion, occurred during pursuit in both the typical and ASD groups. The rate of occurrence of saccades was about the same across the two groups, for saccades that were either in the direction of motion (typical group: mean = 0.90 saccades/s; SD = 0.54; ASD group: mean = 0.72/s, SD = 0.28; F(1,36) = 2.77, n.s.), or opposite to the direction of motion (typical group: mean = 0.22/s, SD = 0.29; ASD group: mean = 0.29/s, SD = 0.18, F(1,36) = 0.87, n.s.). Sizes of saccades, on the other hand, did vary between the groups, with average sizes about twice as large for the ASD group. For saccades in the direction of motion the average size was 30 min arc (SD = 10) for the typical group and 60 min arc (SD = 44) for the ASD group. For saccades opposite to the direction of motion, the average size was 19 min arc (SD = 10) for the typical group and 48 min arc (SD  = 34) for the ASD group. Analysis of variance, using group (ASD v. Typical) and type of motion (cue v. no cue) as factors, confirmed that these large differences in the sizes of saccades across groups were significant, both for saccades in the direction of motion: (F(1,36) = 8.8, p = .0053) and for saccades opposite to the direction of motion (F(1,34) = 11.7, p = .0016). No significant differences were found between saccades sizes with cued vs. uncued motion, and there were no significant interactions between group and the presence of the cue (Tables S1 and S2 in FileS1).

### Maintained fixation

Eye movements measured during maintained fixation of the stationary target were also analyzed because fixation is a task studied frequently in the oculomotor literature [49]. Data were not available from one participant, ASD1, because the horizontal eye trace signal in the fixation trials was lost.

**Figure 5:**
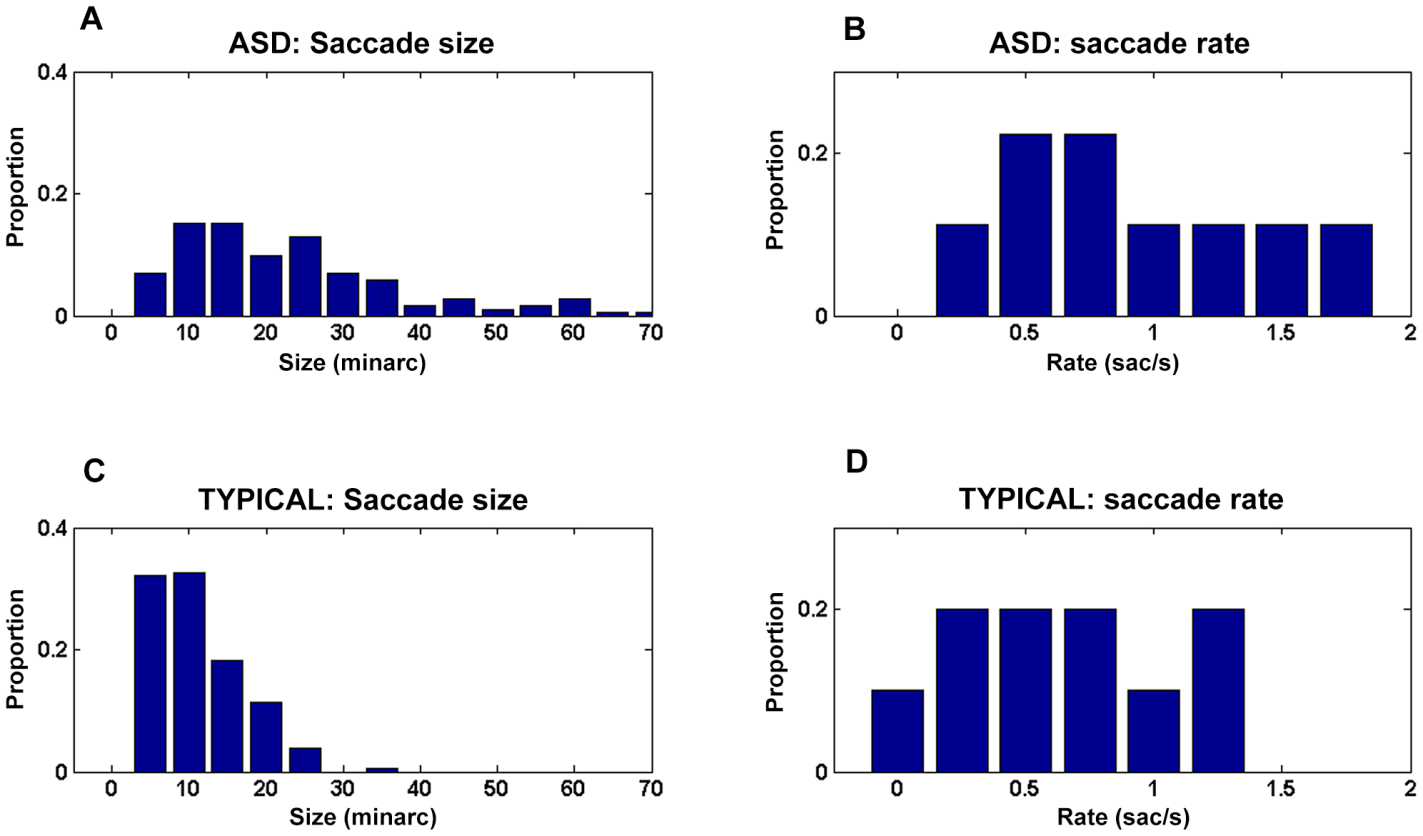
Saccades During Maintained Fixation. (A) Saccade vector sizes in ASD group (B) Saccade rates in ASD group. (C) Saccade sizes in Typical group. (D) Saccade rates in Typical group.

Both groups showed the stereotypical pattern of fixation eye movements (see example in Fig. 2), namely, slow eye movements interspersed with periodic, small saccades [49] with some participants showing saccades very rarely [50]. On average, ASD participants made larger saccades than typicals (Fig. 5 and Table 6), with considerable individual differences in the ASD group. ASD participants also made saccades more frequently. These trends, namely, larger and more frequent saccades during fixation in those with ASD, are consistent with prior reports about saccades in a variety of tasks [33], [35], [46], [47], however, the sample of saccades was small enough, and individual variability large enough, that the differences between groups in either size or rate did not reach significance (Size: *t* = 1.5, df = 17, p = .16, two-tailed. Rate: *t*  =  1.1, df = 17,p = .3,two-tailed).

**Table 6:** Mean size (min arc) and frequency of saccades during maintained fixation.

ASD			Neurotypical		
Participant	Mean size (SD) N	Frequency	Participant	Mean size (SD) N	Frequency
ASD2	20 (16) 28	1.14	TYP1	7 (3) 27	1.08
ASD3	18 (9) 19	0.77	TYP2	10 (2) 11	0.44
ASD4	269 (296) 35	1.67	TYP3	14 (5) 27	1.16
ASD5	13 (4) 10	0.40	TYP4	10 (4) 31	1.24
ASD6	9 (4) 6	0.24	TYP5	85 (98) 5	0.24
ASD7	34 (19) 36	1.44	TYP6	17 (5) 15	0.60
ASD8	60 (27) 11	0.44	TYP7	14 (8) 17	0.68
ASD9	19 (16) 24	0.97	TYP8	10 (2) 5	0.20
ASD10	101 (164) 16	0.80	TYP9	8 (4) 20	0.80
ASD1	not recorded	n/a	TYP10	24 (0) 1	0.04
Group mean (SD)	60.4 (83)	0.87 (.48)	Group mean (SD)	19.9 (23)	0.65 (0.42)

Mean vector size (min arc) and frequency (saccade/s) of saccades of individual participants during maintained fixation.

## Discussion

Individuals with high-functioning ASD were able to produce vigorous anticipatory smooth eye movements in response to visual cues that signaled the future path of a moving target. The velocity of the eye during the anticipatory portion of pursuit was the same as that of a group of typical subjects. The results show that individuals with high-functioning ASD are able to generate accurate predictions about motion in response to cues, and to transform those predictions into smooth oculomotor commands. These results do not support a strong form of theories that posit overall difficulties in generating or using predictions in ASD (for discussion see [38]), and are consistent with the view that the ability to generate and use predictions is intact in ASD [39], [40].

The pursuit task was chosen with the goal of facilitating the formation of accurate predictions about the future path of the target without special effort, instruction or learning. To achieve those ends, we used a highly salient cue whose perceptual characteristics unambiguously signaled the path of motion of the target. The fact that we found anticipatory pursuit on the very first trials in both ASD and typical groups is consistent with the contention that the cue required no special effort or experience, at least not within the laboratory visit.

We cannot discount the possibility that deficits in generating or using predictions in ASD may appear in other oculomotor tasks, such as cases in which the perceptual or semantic links between the cues and the path of motion are different from those we tested. If this were to be the case, however, then any deficits that might emerge would not be in anticipatory smooth pursuit per se, but rather in selected pre-cursors to anticipatory pursuit, such as any processes involved in interpreting the cues or in learning the connection between the cues and the pathway of motion.

It is also possible that deficits in generating anticipatory pursuit eye movements in response to cues may be found in younger participants, or in participants with more severe ASD. Takarae et al. [36], for example, found that those with ASD who also showed language impairments had lower pursuit gain than those without language impairments. Our participants did not demonstrate language impairments.

### Other oculomotor measures

We also examined characteristics of the eye movements in addition to anticipatory pursuit. We found that the saccades during pursuit were larger (by about a factor of two) in the ASD group than in the typical group, although the rate of occurrence of saccades was about the same. We found no statistically reliable differences between ASD and typical participants in either eye velocity after pursuit was underway, or in the characteristics of saccades during maintained fixation, although the direction of the group differences, specifically, the slightly lower pursuit velocities, and the larger and more frequent saccades during fixation in those with ASD, were in line with prior reports [33]–[36], [46], [47].

It is worth noting that findings of lower velocity of smooth pursuit, or larger or more frequent saccades during fixation or during pursuit, are not persuasive indicators of specific oculomotor deficits. This is because these aspects of oculomotor behavior are among those in which individuals have the greatest leverage in choosing how to respond. For example, achieving high smooth pursuit gain (>.9) is not automatic. Pursuit gain can decline due to task strategies, such as a spread of spatial attention to surrounding stationary visual details [51], [52], or lack of sufficient effort [53], [54]. Similarly, the characteristics of saccades during maintained fixation also depend on volition and decisions. Individuals can choose to alter the rate or the sizes of saccades during fixation [49], [50]. Larger or more frequent saccades during fixation could also be an indicator of shifts of attention to surrounding visual details [55], or a reluctance to maintain the line of sight for several seconds in one place when no specific visual task, other than fixation, is required. Thus, conclusions about oculomotor system properties based solely on measures of the gain of pursuit, or the size of frequency of saccades during fixation, must be treated with caution because these properties of oculomotor performance are not necessarily indicators of system deficits, but rather may reflect strategies or decisions about how to perform the task.

### Previous approaches to anticipatory pursuit

The present approach to the study of anticipation in smooth pursuit, which relied on cues, can be compared to previous reports of impairments in anticipatory pursuit based on paradigms that relied on learning or memory. Avila et al. [56], using a method modeled after Wells & Barnes [11] and Barnes et al. [57], studied the changes in pursuit after experience tracking the same constant velocity (ramp) motion for several consecutive presentations. They found that experience tracking the ramps led to both the development of anticipatory pursuit responses prior to the expected time of ramp onset, as well as to higher steady-state smooth pursuit velocity. The results were found for a group of healthy control subjects, as well as for schizophrenic patients, although the eye velocities of the patients were slower. Hong et al. [58] and Moates et al. [59] reported similar findings using a related method involving retinally stabilized targets. They found that both the healthy control subjects and the schizophrenic patients pursued the stabilized targets in patterns that resembled the motion patterns of the unstabilized targets that were pursued in the immediately previous trials, as if the pursuit response reflected the prediction that the patterns of motion seen in the recent past would continue into the future. Once again, however, patients showed lower eye velocities than the controls. The main conclusion of all three studies was that the patients had deficiencies in learning and memory that impaired the generation of anticipatory motor responses.

Impairments in learning and memory would affect the generation of certain types of anticipatory responses that are based either on short-term stores of motion information [10], [11], or on the implicit rule that the patterns of motion seen in the recent past will continue into the future [11], [19], [60]. Anticipatory smooth eye movements that are produced by perceptually salient symbolic cues that are immediately revealing of the motion pathway, on the other hand, are not dependent on learning and memory in that they are not tied to past history [23], and, as demonstrated in the present study, do not require past experience observing or tracking the motion. Thus, the study of anticipatory smooth eye movements produced by symbolic cues can disclose aspects of the ability to generate predictions independently of learning or memory skills. The further study of anticipatory pursuit with cues that signal the path of motion may thus prove to be of value in studying the predictive abilities in both typical and disordered systems, independent of any possible deficits in learning or memory.

### Conclusions and directions for future work

We found that the ability to form predictions and engage in at least one type of predictive motor behavior is present in high-functioning ASD. Anticipatory pursuit in response to perceptually salient cues was elicited on the very first trial, with no special instructions or effort, and with eye velocities that were indistinguishable from the eye velocities found in typical individuals.

These results provide an important gateway to further study of the perception of events and anticipatory behavior in ASD. Such studies could investigate anticipatory pursuit with a variety of cues, such as cues with arbitrary links to the motion pattern (e.g., red discs move left; green discs move right), or cues whose links to the motion require interpretations of the scene or of the ongoing behavior of others (e.g., the direction of motion of a diver leaning over a board, or the direction of motion of a ball about the leave the hand of a pitcher). Once the ability to generate anticipatory pursuit is demonstrated, the study of the anticipatory responses with a variety of types of cues may provide a window into the perceptual or cognitive processes that underlie the interpretation of events in natural environments or social situations.

## Supporting Information

File S1 **Tables S1 and S2. Table S1.** ANOVA statistics on saccadic eye movements during steady state pursuit, for saccades in the direction of target motion. **Table S2.** ANOVA statistics on saccadic eye movements during steady state pursuit, for saccades opposite to the direction of target motion.
(DOC)
