# Supplementary material for: Anticipatory Smooth Eye Movements in Autism Spectrum Disorder
Source: PLoS One. 2013 Dec 23;8(12):e83230. doi: 10.1371/journal.pone.0083230 (PMC3871521; doi:10.1371/journal.pone.0083230)
Supplement: File S1 — Tables S1 and S2. Table S1. ANOVA statistics on saccadic eye movements during steady state pursuit, for saccades in the direction of target motion. Table S2. ANOVA statistics on saccadic eye movements during steady state pursuit, for saccades opposite to the direction of target motion. (DOC) [file pone.0083230.s001.doc]

**Table S1. ANOVA statistics on saccadic eye movements during steady state pursuit, for saccades in the direction of target motion**

| **Source** | **Sum of Squares** | **df** | **Mean Squares** | ***F*** | ***p*** |
| --- | --- | --- | --- | --- | --- |
| **ASD/Neurotypical** | 9253.2 | 1 | 9253.2 | 8.81 | 0.0053 |
| **Cued/Uncued** | 35.5 | 1 | 35.5 | .03 | 0.8551 |
| **Condition by Cueing** | 490.8 | 1 | 490.8 | 0.47 | 0.4986 |
| **Error** | 37,807.2 | 36 | 1050.2 |  |  |
| **Total** | 47,586.7 | 39 |  |  |  |

**Table S2. ANOVA statistics on saccadic eye movements during steady state pursuit, for saccades opposite to the direction of target motion**

| **Source** | **Sum of Squares** | **df** | **Mean Squares** | ***F*** | ***p*** |
| --- | --- | --- | --- | --- | --- |
| **ASD/Neurotypical** | 8228.5 | 1 | 8228.5 | 11.7 | 0.0016 |
| **Cued/Uncued** | 41.8 | 1 | 41.8 | 0.06 | 0.8088 |
| **Condition by Cueing** | 222.2 | 1 | 222.2 | 0.32 | 0.5777 |
| **Error** | 26,902.9 | 34 | 703.03 |  |  |
| **Total** | 32,547.7 | 37 |  |  |  |
